# Signatures of selection in Angus and Hanwoo beef cattle using imputed whole genome sequence data

**DOI:** 10.3389/fgene.2024.1368710

**Published:** 2024-08-02

**Authors:** Muhammad Yasir Nawaz, Rodrigo Pelicioni Savegnago, Dajeong Lim, Seung Hwan Lee, Cedric Gondro

**Affiliations:** ^1^ Department of Animal Science, Michigan State University, East Lansing, MI, United States; ^2^ Genetics and Genome Sciences Graduate Program, Michigan State University, East Lansing, MI, United States; ^3^ Division of Animal and Dairy Science, Chungnam National University, Daejeon, Republic of Korea

**Keywords:** signatures of selection, Hanwoo, Angus, WGS, beef cattle

## Abstract

In this study, we detected signatures of selection in Hanwoo and Angus beef cattle using allele frequency and haplotype-based methods based on imputed whole genome sequence variants. Our dataset included 13,202 Angus animals with 10,057,633 imputed SNPs and 10,437 Hanwoo animals with 13,241,550 imputed SNPs. The dataset was subset down to 6,873,624 SNPs in common between the two populations to identify within population (runs of homozygosity, extended haplotype homozygosity) and between population signals of selection (allele fixation index, extended haplotype homozygosity). Assuming these selection signals were complementary to each other, they were combined into a decorrelated composite of multiple signals to identify regions under selection for each of the breeds. 27 genomic regions spanning 25.15 Mb and harboring 360 genes were identified in Angus on chromosomes 1,3, 4, 5, 6, 7, 8, 12, 13, 14, 16, 20, 21 and 28. Similarly, in Hanwoo, 59 genes and 17 genomic regions spanning 5.21 Mb on chromosomes 2, 4, 5, 6, 7, 8, 9, 10, 13, 17, 20 and 24 were identified. Apart from a small region on chromosome 13, there was no major overlap of selection signals between the two breeds reflecting their largely different selection histories, environmental challenges, breeding objectives and breed characteristics. Positional candidate genes identified in selected genomic regions in Angus have been previously associated with growth, immunity, reproductive development, feed efficiency and adaptation to environment while the candidate genes identified in Hanwoo included important genes regulating meat quality, fat deposition, cholesterol metabolism, lipid synthesis, neuronal development, and olfactory reception.

## 1 Introduction

Natural selection is an adaptive response to the environment a population inhabits, which drives its evolutionary changes by favoring traits that are advantageous and increases their prevalence in the population. Very recently, at least on an evolutionary scale, human driven artificial selection has also become a primary driver of changes in populations by exerting selective pressure on traits of human interest. A prime example of artificial selection is dog breeding: dogs have been bred for various desireable characteristics which led to a wide variety of breeds from the tiny Chihuahua to the massive Great Dane. Such selection processes change allele frequencies in populations and leave traceable marks across the genome. Genomic regions under selective pressure can be identified by their allele frequency distributions, measures of linkage disequilibrium between loci and the structure of their haplotypes. Identification of these genetic patterns or *signatures of selection* (SOS) help us understand the underlying biological processes of adaptation in different environments and provide insights into the domestication history of agricultural species. They can also help us identify genes or genomic regions that regulate the phenotypic expression of traits of economic importance. For example, studies of signatures of selection have been used to identify genes that regulate coat color and body size in dogs (Pollinger et al., 2005; Sutter et al., 2007), stature in horses (Makvandi-Nejad et al., 2012), and body temperature maintenance under cold stress in cattle (Igoshin et al., 2019). Randhawa et al. (2016) published a meta-assembly of selection signatures in cattle genome by combining results from various studies. They found that a number of selection hotspots have been identified in European cattle but studies on major cattle groups like Zebu, African and Composite cattle have been few. They also observed that most of the selection signals were unique for each breeds while some were shared across breeds. The most prominent peaks were observed in genes of known major effects like coat color, polled locus and muscle hypertrophy.

Various methods have been proposed to identify genomic signatures of selection which can be broadly classified into two main categories: within population measures for a single population (e.g., runs of homozygosity and integrated haplotype score) or between population measures that compare two or more populations (e.g., fixation index and cross-population extended haplotype homozygosity). Each of these test statistics explore unique facets of the genomic architecture of populations but they are not necessarily consistent with each other. Inconsistencies between selection sweeps are observed not only due to the inherent differences in statistical methodologies but also due to differential sensitivity to sampling, demographic history and linkage disequilibrium between loci (Ewing and Jensen, 2016). Therefore, some studies take a more conservative approach and only focus on the regions that are common across different measures, albeit at the risk of not identifying a proportion of the relevant signals in the process. An alternative approach is to consider the selection signals from different methods as complimentary to each other (Ma et al., 2015) and combine them to get a composite score (Randhawa et al., 2014). Various methods to combine individual signals have already been proposed in the literature (Grossman et al., 2010; Utsunomiya et al., 2013b; Randhawa et al., 2014; Ma et al., 2015). Initial approaches to combine the signals did not account for the covariance structure between signals but Ma et al. (2015) suggested a new approach to calculate a decorrelated composite of multiple signals (DCMS) that adjusted for correlations between signals and was more powerful to detect selected regions in the genome.

This study focused on the identification of signatures of selection in Angus and Hanwoo cattle. Both are beef cattle breeds, but they have been subjected to entirely different selection pressures and have different genetic population structures, body characteristics, domestication history, beef quality and breeding program objectives. Hanwoo are Korean taurine cattle, more related to Asian taurine cattle like Japanese Wagyu than to western taurine cattle breeds (Angus, Hereford, etc.) (Lee et al., 2014). Hanwoo have a smaller stature than Angus, but its beef is popular for its juiciness, high levels of marbling, and unique flavor (Cho et al., 2010); which, similarly to Wagyu, attracts a market premium. Hanwoo was historically a draft breed kept by small holder farmers which accounted for more than 99% of the farms in Korea until 1985. Hanwoo steers are typically kept up to 30–32 months of age to improve the marbling score. In 1960s, various breed improvement initiatives were taken in Korea. The recent advances in management of beef production have also led to an increase in the size of beef operations in Korea. Currently, the selection index of the *Korean Proven Bulls* program is mainly driven by 4 traits–marbling score (MS), carcass weight (CWT), eye muscle area (EMA) and back fat thickness (BF). Consequently, Hanwoo have shown considerable improvement in beef quality. Angus, on the other hand, are European taurine cattle that originated from Scotland. Angus have been intensively selected for growth, stature and feed intake in the 20th century and have become the most common beef cattle in the world. Angus are characterized by their high muscularity, fast growth rate, medium height, and moderate levels of intramuscular fat (Albertí et al., 2008). In contrast to Hanwoo, different selection indices are used in Angus cattle breeding programs worldwide depending on the type of beef production operation and its breeding objectives. The average age at slaughter varies between 12 and 20 months depending on whether the calves are weaned and sent directly to a feeding facility to be finished for slaughter or they are grown on grass pastures at first, followed by a high-energy diet for a short period of time (100–120 days) before slaughter. Therefore, due to stark differences in evolutionary origin, artificial selection, farming systems, and body characteristics, differences in genomic landscape between them may point to genetic basis of adaptive traits and meat production.

The objectives of this study were to identify genome wide signals of selection in Angus and Hanwoo beef cattle using imputed whole genome sequence (WGS) data. We used imputed whole genome sequence data for this analysis to get a higher resolution of selected genomic regions. We also combined individual selection measures to obtain a decorrelated composite of multiple signals (DCMS) for identification of selected genomic regions. These signatures of selection were then mapped to the ARS-UCD1.2 reference assembly to identify candidate genes located in these regions. We also highlight important genes related to meat production and quality.

## 2 Materials and methods

### 2.1 Genotype data

Imputed whole genome genotypes of 10,437 Hanwoo animals (13,241,550 SNPs) and 13,202 Angus animals (10,057,633 SNPs) were utilized for this analysis. Respectively, the Hanwoo and Angus data consisted of 9,160 and 11,632 animals genotyped on 50k arrays (Illumina Bovine SNP50 BeadChip; Illumina, San Diego, CA), 1,704 and 1,236 animals genotyped on 700k arrays (777k SNP, Illumina Bovine HD Beadchip, Illumina, San Diego, CA), and 203 and 334 reference animals with whole genome sequence (WGS) data. All Hanwoo animals originated from commercial farms in Korea while Angus data was collected from commercial farms primarily in the US. Sequence analysis was performed using integrated variant discovery pipeline (https://github.com/rodrigopsav/IVDP) to call variants. The key steps in the pipeline include read trimming and adapter removal by trimmomatic, read alignment to ARS-UCD1.2 *Bos taurus* assembly using bwa-mem2, duplicated read marking by sambamba-markdup, base quality recalibration using GATK BaseRecalibratorSpark and ApplyBQSRSpark and variant calling using GATK HaplotypeCaller. Sequenced animals were used a reference to impute genotype data of their respective breeds. Eagle software version 2.3.2 and Minimac3 was used for phasing and imputation respectively. Details on quality control, WGS pipeline and imputation accuracies for Hanwoo were previously reported in Nawaz et al., 2022. Finally, Imputed whole genome data was subset down to the *6,873,624* SNPs that were common between the two breeds to calculate across population measures of selection.

### 2.2 Analysis

We performed principal component analysis on the combined dataset containing all Angus and Hanwoo animals using plink 1.9 to evaluate population structure in the data. Various selection signals were calculated as explained below.

#### 2.2.1 Within population measures

Runs of homozygosity (ROH) are defined as long continuous homozygous genomic regions that are assumed to be identical DNA segments inherited by descent from a common ancestor, and that serve as an indicator of genomic autozygosity, consanguinity, selection, and population size reduction. ROH detection was done using the *homozyg* function in plink using the default parameters except for the number of SNPs in a scanning window (*homozyg-window-snp*) which was increased to 100 instead of default 50 SNPs because of the high density of SNPs in the sequence data. Default values were used for all the other required parameters in *homozyg* function.

To identify ROH islands, we calculated the autozygosity of each SNP by taking the proportion of individuals in which a SNP was identified within a ROH region.

Integrated haplotype score (iHS) aims to identify genomic regions that were under recent positive selection based on the relationship between an allele’s frequency and the extent of linkage disequilibrium around it. iHS was calculated (Voight et al., 2006) based on extended haplotype homozygosity (EHH) values (Sabeti et al., 2002) calculated using the program *hapbin* (Maclean et al., 2015). Due to the high dimensionality of our data and computational limitations of the software, the analysis was performed by dividing both Hanwoo and Angus datasets into seven and 14 bins containing 1491 and 943 animals per bin, respectively. The correlation of iHS between sample bins ranged from 0.86 to 0.93. Final values of iHS were calculated by taking the average of iHS values from the data bins. Absolute values of iHS were smoothed out in windows of 1,001 SNPs to identify regions under recent positive selection.

#### 2.2.2 Across population measures

Fixation Index (F_ST_) is a measure of population differentiation. It represents the proportion of total genetic variance that exists within a sub population. Allele frequencies of Angus and Hanwoo datasets were calculated using *freq* function in *plink*. Average of Angus and Hanwoo allele frequencies were used as the baseline allele frequency ( p ) and genetic variances ( $\left.p*\left(1-p\right)\right)$ . Finally, F_ST_ was calculated for each SNP by taking squared deviation of allele frequency in a breed from the baseline frequency divided by allelic variance (Weir and Cockerham, 1984):

$$F_{ST}=\frac{\sigma^{2}}{p\left(1-p\right)}$$

To identify prominent genomic regions, F_ST_ was smoothed in windows of 1,001 SNPs using *runmed* function in base R.

Across population extended haplotype homozygosity (XPEHH) (Sabeti et al., 2007) is another population differentiation-based test that is used to detect selective sweeps in which selected regions are close to fixation in one population but remain polymorphic in another population. For XPEHH, we compared the two breeds under study (Angus and Hanwoo) directly against each other to identify regions that were differentially selected between populations. We used the *hapbin* software (Maclean et al., 2015) to perform this analysis with the *xpehh* function.

#### 2.2.3 Decorrelated composite of multiple signals (DCMS)

In order to combine the several test statistics, we used the method suggested by Ma et al. (Ma et al., 2015) that takes into account correlations between signals to calculate a decorrelated composite of multiple signals (DCMS) based on their *p* values. Firstly, fractional ranks of autozyosity and absolute values of ROH, iHS, FST and XPEHH were used to calculate their *p* values using *stat_to_p-value* function in R package *MINOTAUR* (with parameters *two.tailed = FALSE, right.tailed = TRUE*). Then, a pairwise correlation matrix was created between absolute values of the signals. This matrix was used as an input to DCMS function in MINOTAUR to calculate raw DCMS scores as follows (Ma et al., 2015):

$$DCMS=\sum_{t=1}^{n}\frac{\left(log\;\frac{1-p_{lt}}{p_{lt}}\right)}{\sum_{i=1}^{n}\left|r_{it}\right|}$$

$p_{lt}$ was the *p*-value of individual selection measures and $r_{it}$ was the Pearson correlation between two measures. Finally, *p* values of raw *DCMS* scores were calculated by *pnorm* function using empirical mean and standard deviation. Multiple test correction was done by calculating false positive rate (*FDR*) using *p.adjust* function in base R with *method= ‘BH’.* SNPs having adjusted *p*-values (*q*) less than 0.05 were deemed to be significant. Adjacent significant SNPs (located less than 1 MB apart) were combined to identify regions under selection by a custom script in R.

#### 2.2.4 Functional annotation of signatures of selection

A *Bos taurus* gene annotation dataset which included positional information for all known bovine genes (n = 27,900) mapped to the latest bovine assembly (*ARS_UCD1.2*) was downloaded from ensemble with *BIOMART*. Significant genomic regions were mapped to genes using the *GenomicRanges* package in R (Lawrence et al., 2013).

## 3 Results

**FIGURE 1:**
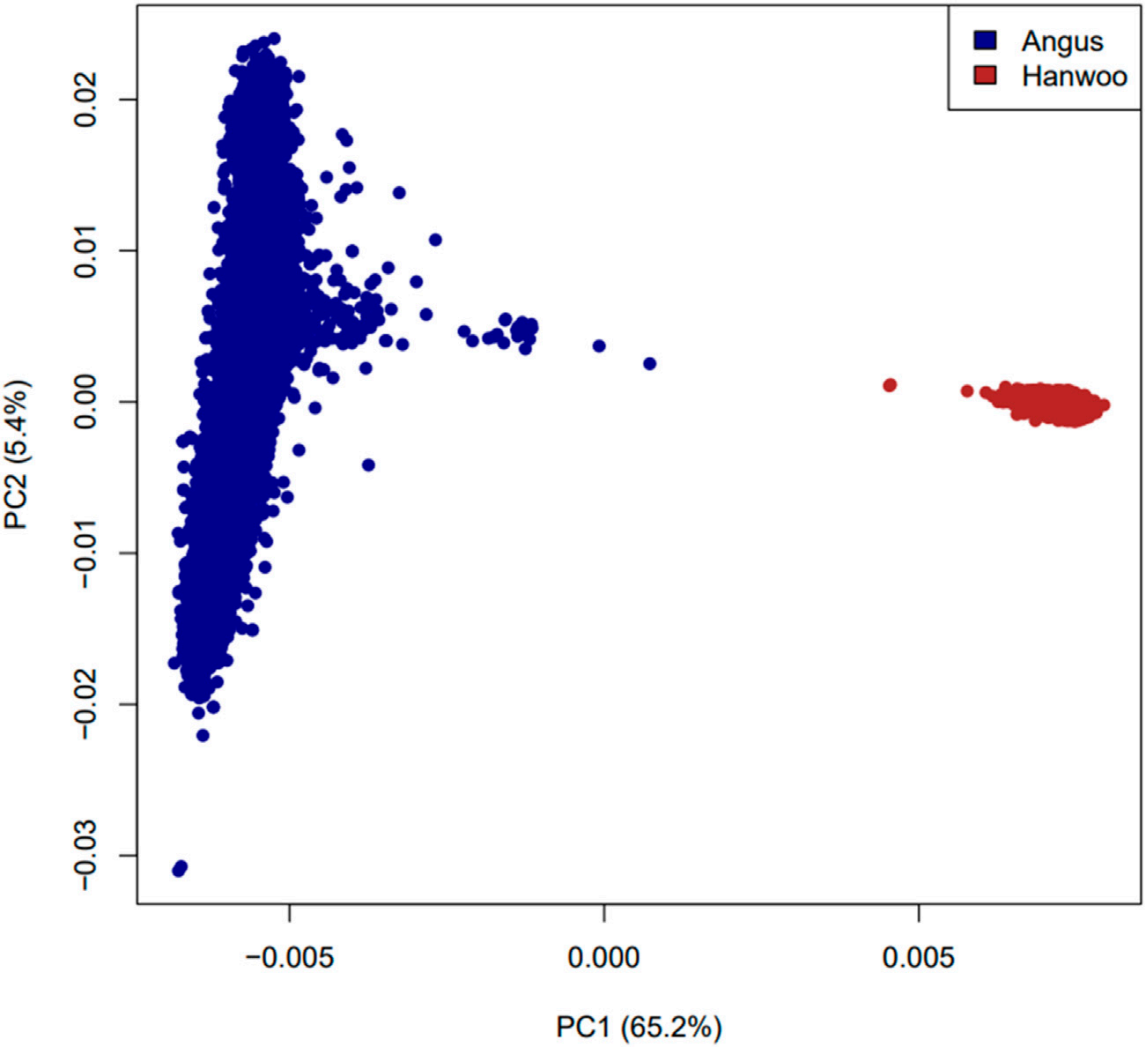
Plot of first two principal components based on a relationship matrix constructed from 6,873,624 SNPs common between Angus and Hanwoo.

The observed heterozygosity in Angus and Hanwoo cattle was 0.30 and 0.31 respectively. Principal component analysis revealed that Angus and Hanwoo animals clearly clustered separately from each other in tight clusters (Figure 1). The first principal component separated the two populations and accounted for 65.1% of genomic variation in the dataset. The second principal component captured variation in Angus animals which accounted for only 5.4% of the total genomic variation in the dataset. These results indicate that majority of the genomic variation in the dataset can be explained by the differences in genomic architecture of the two breeds.

### 3.1 Within population measures

ROH: The mean number of ROH detected per animal was higher in Angus (88.7 ± 18.50) as compared to Hanwoo (12.5 ± 8.4) (Table 1). The median length of ROH regions was also higher in Angus (1,565 BP) as compared to Hanwoo (1,384 BP). However, the proportion of ROH regions longer than 5 MB was higher in Hanwoo (12.5%) than Angus (7.1%). Therefore, Hanwoo had fewer ROH regions, but they were longer than in Angus suggesting a comparatively more recent selection in Hanwoo. Mean genome wide autozygosity was higher in Angus (0.08) as compared to Hanwoo (0.01). The highest peak for Hanwoo was observed on CHR 7 (BP 50280340) and smaller peaks were observed on CHR 2, 12, 23, 24 and 29. In Angus, the strongest signal was observed on CHR 13 (BP 63,854,457). Other significant peaks were also identified on CHR 8 and 14.

**TABLE 1:** Summary of results from runs of homozygosity analysis for Hanwoo and Angus cattle.

Parameter	Hanwoo	Angus
Total SNPs	13241550	10057633
Total animals	10437	13202
Percentage of animals having ROH	99.7	99.9
Total number of ROH regions	129778	1169509
Mean number of ROH per animal	12.5	88.7
SD of number of ROH per animal	8.4	18.501
Minimum number of ROH per animal	1	1
Maximum number of ROH per animal	440	270
Mean length of ROH regions in KB	3024	2381.845
SD of length of ROH regions	5089.125	2965.864
Median length of ROH regions	1384	1565
Maximum length of ROH	123720	120023
Minimum length of ROH	1000	1000
No of ROH per animal	12.46	88.67
ROH 1–5 mb	113515 (87.5%)	1087433 (92.9%)
ROH 5–10 mb	8369 (6.5%)	56661 (4.9%)
ROH 10–15 mb	3616 (2.8%)	13443 (1.1%)
ROH 15–20 mb	1718 (1.3%)	5491 (0.4%)
ROH 20–25 mb	1017 (0.8%)	2828 (0.2%)
ROH 25–30 mb	614 (0.5%)	1527 (0.1%)
ROH >30 mb	929 (0.7%)	2126 (0.2%)

iHS: Genome wide distribution of absolute iHS values was similar in Hanwoo and Angus with a mean of 0.31 and 0.30 respectively. Absolute value of iHS indicated genomic regions with unusually long haplotypes on chromosomes 1, 5, 6, 8, 10, 11, 13, 16, 17, 20, 23, 24 and 29 in Angus harboring 13,009 significant SNPs. The strongest signal was detected on CHR 16 (rs208273139) at 40,588,657 BP. In Hanwoo, the strongest signal was observed on chromosome 2 at (rs207720085) 82,874,034 BP. Other peaks were observed on chromosomes 1, 2, 3, 5, 6, 7, 8, 9, 14, 17, 20, 25 and 26 harboring 13,030 significant SNPs. Correlation between iHS values of Angus and Hanwoo was 0.016 indicating differences in the regions of selection sweeps between the two breeds.

We also observed that ROH and iHS were significantly correlated (R = 0.252, 95% confidence interval 0.251–0.253) in Hanwoo and Angus (R = 0.286, 95% interval 0.286–0.287).

### 3.2 Across population measures

Fixation index F_ST_: SNPs with an FST value in the top 0.2% were identified on 18 out of 29 autosomes indicating widespread allele frequency differences between breeds. CHR 4 contained the highest number of significant SNPs (n = 1,577) followed by CHR 8 (n = 1,464) and CHR 5 (n = 1,256). The most significant SNP (rs209900249) was observed on CHR 4 position 69,682,473. Other prominent FST hotspots were observed on CHR 1, 2, 3, 6, 7, 9, 10, 13, 14, 16, 18, 20, 21, 28, and 29.

Across population extended haplotype homozygosity (XPEHH): 13,004 SNPs with top 0.2% XPEHH values were located on CHR 3 (n = 2,216), 8 (n = 4,826), 13 (n = 4,115) and 14 (n = 1,847). The most significant peak was observed on CHR 13 at position 62,594,885 (rs207508467).

We also observed that the two measures of across population measures were significantly correlated, Pearson correlation R = 0.2956 and a 95% confidence interval 0.295–0.296.

### 3.3 Decorrelated composite of multiple signals (DCMS)

**FIGURE 2:**
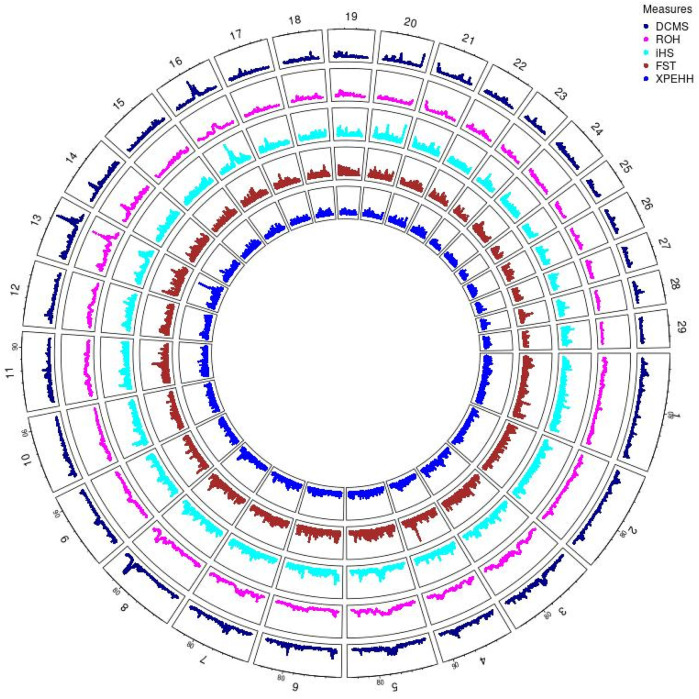
Circos plot of *p*-values for genome wide signatures of selection in Angus cattle.

**FIGURE 3:**
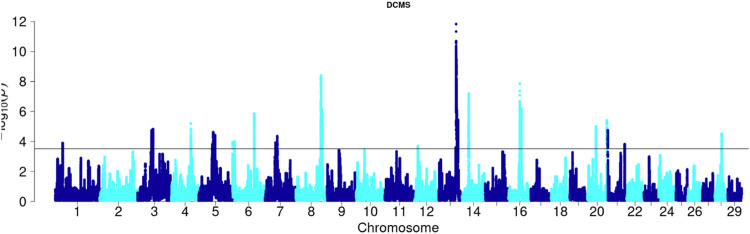
Manhattan plot of DCMS *p*-values in Angus cattle. Horizontal black line indicates the significance cut off (0.05 FDR).

Angus: A total of 39,898 SNPs were identified with significant *p*-values. Genic SNPs accounted for 27.49% of all the significant SNPs. 27 significant genomic regions were identified using the DCMS adjusted *p*-value (q value) cutoff of 0.05. The mean length of selected regions was 931.613 Kb (±1,255.33) while their total length was 25.153 Mb. The significant genic regions mapped to CHR 1,3, 4, 5, 6, 7, 8, 12, 13, 14, 16, 20, 21, and 28 (Figures 2, 3) that harbor 360 genes (Table 2). The most significant genomic selection signal was observed on CHR 13 where 91 genes were found spread across 3 distinct regions.

**TABLE 2:** Genomic regions under selection in Angus cattle identified by DCMS q values ≤ 0.05 and genes identified in those regions.

Start	End	CHR	No of genes	Genes
26683714	26745320	1	0	None
50460996	53793201	3	35	MTF2 DIPK1A RPL5 SNORA66 SNORD21 U6 EVI5 ENSBTAG00000055274 5S_rRNA GFI1 RPAP2 GLMN C3H1orf146 BTBD8 ENSBTAG00000040248 EPHX4 BRDT ENSBTAG00000054884 ENSBTAG00000047443 TGFBR3 CDC7 HFM1 ENSBTAG00000054082 ENSBTAG00000046077 ENSBTAG00000055150 ZNF644 bta-mir-2285b-2 BARHL2 ZNF326 LRRC8D bta-mir-2285k-5 ENSBTAG00000050182 LRRC8C LRRC8B ENSBTAG00000038625
55238880	55468638	3	3	PKN2 ENSBTAG00000051499 ENSBTAG00000051844
69475528	69894433	4	6	7SK SNX10 CBX3 HNRNPA2B1 NFE2L3 MIR148A
48288996	48752060	5	4	MSRB3 LEMD3 WIF1 U6
52011261	52117527	5	1	TAFA2
53692271	54424033	5	3	SLC16A7 ENSBTAG00000055198 ENSBTAG00000053531
58055499	59307814	5	42	U6 ENSBTAG00000047825 ENSBTAG00000052093 ENSBTAG00000049329 ENSBTAG00000051156 ENSBTAG00000046778 ENSBTAG00000048295 ENSBTAG00000054507 ENSBTAG00000050480 ENSBTAG00000051165 ENSBTAG00000051462 ENSBTAG00000049219 ENSBTAG00000051274 ENSBTAG00000048779 OR6C76 OR6C75 ENSBTAG00000049581 ENSBTAG00000049184 ENSBTAG00000048408 ENSBTAG00000024691 ENSBTAG00000051265 ENSBTAG00000050381 ENSBTAG00000049213 ENSBTAG00000054097 ENSBTAG00000049016 ENSBTAG00000045922 ENSBTAG00000048168 ENSBTAG00000053702 ENSBTAG00000054733 ENSBTAG00000049913 ENSBTAG00000051198 ENSBTAG00000002913 ENSBTAG00000051990 ENSBTAG00000048864 ENSBTAG00000046446 ENSBTAG00000049753 ENSBTAG00000054193 OR10A7 ENSBTAG00000049751 ENSBTAG00000053229 ENSBTAG00000053772 ENSBTAG00000037629
1155763	1320935	6	1	ENSBTAG00000051456
8724140	8824920	6	0	None
78535547	78939028	6	0	None
38009575	38227430	7	8	FAF2 RNF44 CDHR2 GPRIN1 SNCB EIF4E1B TSPAN17 UNC5A
44026848	44466715	7	22	ENSBTAG00000012150 MEX3D MBD3 UQCR11 TCF3 ONECUT3 ATP8B3 REXO1 KLF16 ABHD17A ENSBTAG00000050118 SCAMP4 CSNK1G2 bta-mir-6120 BTBD2 SOWAHA SHROOM1 GDF9 UQCRQ LEAP2 AFF4 U6
89565309	94976398	8	42	5S_rRNA ENSBTAG00000052296 NXNL2 SPIN1 ENSBTAG00000051928 ENSBTAG00000054632 CDK20 FBXW12 ENSBTAG00000021235 MSANTD3 TMEFF1 CAVIN4 PLPPR1 5S_rRNA ENSBTAG00000025760 MRPL50 ZNF189 ALDOB PGAP4 RNF20 GRIN3A ENSBTAG00000050971 ENSBTAG00000030953 CYLC2 U6 SMC2 ENSBTAG00000047350 ENSBTAG00000013445 ENSBTAG00000050829 ENSBTAG00000052864 ENSBTAG00000053491 ENSBTAG00000016173 ENSBTAG00000050464 ENSBTAG00000000145 ENSBTAG00000052019 OR13C3 ENSBTAG00000049256 OR13C8 ENSBTAG00000048409 NIPSNAP3A ABCA1 SLC44A1
12701319	12716083	12	1	TNFSF11
61100092	61130961	13	12	ENSBTAG00000052743 DEFB121 DEFB122A DEFB122 DEFB123 DEFB124 REM1 HM13 bta-mir-12010 ID1 COX4I2 BCL2L1
62482399	65519468	13	71	BPIFB4 BPIFA2A ENSBTAG00000031375 BPIFA2C ENSBTAG00000011704 BPIFA2B ENSBTAG00000031373 BPIFA3 BPIFA1 BPIFB1 BPIFB5 CDK5RAP1 ENSBTAG00000031354 SNTA1 ENSBTAG00000010131 ENSBTAG00000053051 ENSBTAG00000053797 NECAB3 C13H20orf144 E2F1 PXMP4 ZNF341 CHMP4B RALY EIF2S2 ASIP AHCY ENSBTAG00000050108 ENSBTAG00000046623 ITCH DYNLRB1 MAP1LC3A PIGU TP53INP2 NCOA6 GGT7 ACSS2 GSS MYH7B bta-mir-499 TRPC4AP EDEM2 PROCR MMP24 EIF6 FAM83C UQCC1 ENSBTAG00000053266 GDF5 ENSBTAG00000052250 CEP250 ENSBTAG00000030976 ERGIC3 ENSBTAG00000053187 SPAG4 CPNE1 RBM12 NFS1 ROMO1 RBM39 ENSBTAG00000053775 PHF20 5S_rRNA SCAND1 CNBD2 ENSBTAG00000052997 ENSBTAG00000053403 EPB41L1 ENSBTAG00000050801 AAR2 DLGAP4
67831405	69506639	13	8	FAM83D ENSBTAG00000044690 DHX35 U6 ENSBTAG00000049087 ENSBTAG00000050378 ENSBTAG00000048871 MAFB
22710076	24757731	14	24	XKR4 TMEM68 TGS1 LYN RPS20 ENSBTAG00000045097 U1 MOS PLAG1 CHCHD7 ENSBTAG00000054153 SDR16C5 SDR16C6 PENK U6 BPNT2 FAM110B ENSBTAG00000047136 ENSBTAG00000051748 UBXN2B CYP7A1 U1 SDCBP NSMAF
40378981	41205611	16	9	TNFSF18 ENSBTAG00000052047 ENSBTAG00000053302 TNFSF4 ENSBTAG00000020550 AADACL4 DHRS3 VPS13D SNORA59A
42527352	44175641	16	32	MTOR ANGPTL7 EXOSC10 SRM MASP2 TARDBP CASZ1 PEX14 DFFA ENSBTAG00000045105 CORT CENPS PGD ENSBTAG00000048790 ENSBTAG00000048747 ENSBTAG00000054239 U6 UBE4B RBP7 NMNAT1 LZIC CTNNBIP1 CLSTN1 PIK3CD U6 5S_rRNA U6 TMEM201 SLC25A33 ENSBTAG00000049485 SPSB1 H6PD
45628162	46172668	16	3	ENSBTAG00000048839 ENSBTAG00000051176 CAMTA1
31142802	31450979	20	11	ENSBTAG00000033187 NNT PAIP1 ENSBTAG00000049623 C20H5orf34 TMEM267 CCL28 HMGCS1 ENSBTAG00000048672 NIM1K ENSBTAG00000042376
69997413	70839780	20	8	ENSBTAG00000050065 IRX2 U6 ENSBTAG00000054006 5S_rRNA IRX4 NDUFS6 ENSBTAG00000050317
2650859	3126131	21	2	ATP10A U6
62909681	63080951	21	3	5S_rRNA ENSBTAG00000049199 ENSBTAG00000052737
25323298	25520626	28	9	DDX21 KIFBP U6 SRGN ENSBTAG00000042264 ENSBTAG00000051145 VPS26A SUPV3L1 HKDC1

Some of the notable genes identified in significant genomic regions were associated with body size and stature (PLAG1, CHCHD7, RPS20, LYN), growth and feed intake (TMEM68, TGS1, LYN, XKR4), growth differentiation factor (GDF5), feed efficiency (OR6C76, PIK3CD), embryonic growth and reproductive development (NMNAT1), immunity related to tropical adaptation (SLC25A33, SPSB1), immune response and immune regulation (PIK3CD), pigmentation and adaptation to environment (ASIP). A complete list of all the regions and genes identified is shown in Table 2.

**FIGURE 4:**
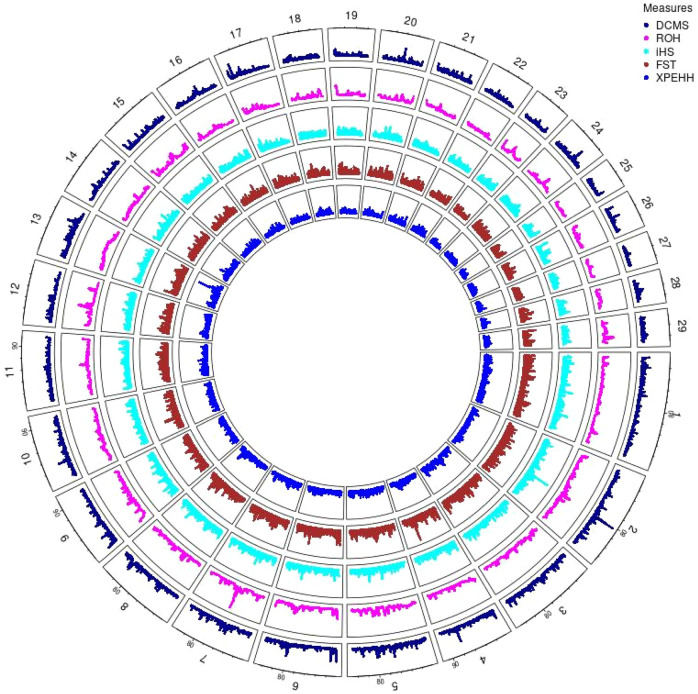
Circos plot of *p*-values for genome wide signatures of selection in Hanwoo cattle.

**FIGURE 5:**
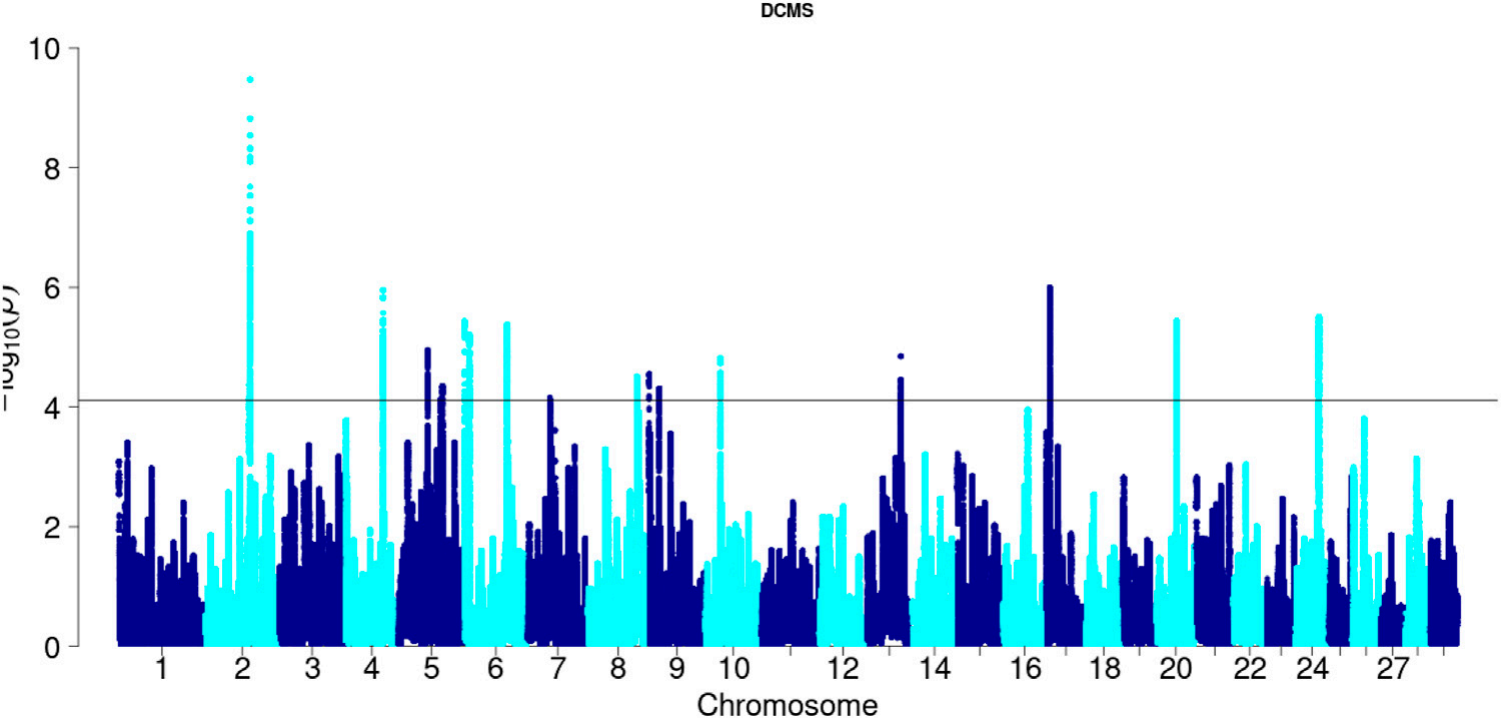
Manhattan plot of DCMS *p*-values in Hanwoo cattle. Horizontal black line indicates the significance cut off (0.05 FDR).

Hanwoo: A total of 10,162 SNPs were found in significant hotspots of selection using FDR cut off value of 0.05 on adjusted DCMS *p* values (q value). Out of these only 2,095 (20.6%) SNPs were located in genes. Significant SNPs were used to identify 17 significant genomic regions. The mean length of the selected regions was 306.27 kb (± 337.43) while their total length was 5.21 Mb. Significant genomic regions mapped to CHR 2, 4, 5, 6, 7, 8, 9, 10, 13, 17, 20, and 24 (Figures 4, 5) which harbor 59 genes (Table 3).

**TABLE 3:** Genomic regions under selection in Hanwoo cattle identified by DCMS q values ≤ 0.05 and genes identified in those regions.

Start	End	Chr	No of genes	Genes
81860076	82963443	2	1	ENSBTAG00000048361
69605816	69985802	4	5	SNX10 CBX3 HNRNPA2B1 NFE2L3 MIR148A
53668560	53873255	5	3	SLC16A7 ENSBTAG00000055198 ENSBTAG00000053531
77883587	77916175	5	1	RESF1
80625444	80681767	5	0	None
1092999	1344929	6	1	ENSBTAG00000051456
9935965	10067004	6	0	None
78629307	79704653	6	3	ENSBTAG00000054580 5S_rRNA TECRL
41006911	41016811	7	9	ENSBTAG00000039484 OR2G2 ENSBTAG00000030735 OR2G3 ENSBTAG00000039804 ENSBTAG00000052311 ENSBTAG00000054452 OR6F1 ENSBTAG00000045691
90427692	90752832	8	4	TMEFF1 CAVIN4 PLPPR1 5S_rRNA
1692932	1844248	9	0	None
20087216	20220978	9	1	BCKDHB
27861580	27896822	10	10	ENSBTAG00000050516 ENSBTAG00000038188 ENSBTAG00000013255 ENSBTAG00000053839 ENSBTAG00000047465 ENSBTAG00000051986 ENSBTAG00000053279 ENSBTAG00000003549 OR4F15 ENSBTAG00000052056
62709174	62826868	13	10	ENSBTAG00000011704 BPIFA2B ENSBTAG00000031373 BPIFA3 BPIFA1 BPIFB1 BPIFB5 CDK5RAP1 ENSBTAG00000031354 SNTA1
7070755	7207418	17	2	LRBA MAB21L2
37138810	37552209	20	5	bta-mir-2360 CPLANE1 NIPBL ENSBTAG00000050782 SLC1A3
43661886	44310133	24	4	MC2R ENSBTAG00000048673 ENSBTAG00000046153 SETBP1

The most significant genomic region was on CHR 2 between BP 81860076 and 82963443 BP where only 1 gene was identified (ENSBTAG00000048361). The greatest number of SNPs mapped to a gene on CHR 17 that plays important role in immunity (LRBA). An important region on CHR *24 (BP 43384983–44317964)* was identified that contained genes (*e.g., MC2R*) regulating fat deposition and meat quality. Other genes identified were previously associated with important roles in brain development (CPLANE1), developmental regulation (NIPBL), breakdown of amino acids (BCKDHB), olfactory reception (OR6F1). A complete list of all the regions and genes identified has been provided in Table 3. Interestingly, none of the significantly selected regions were common between Hanwoo and Angus.

## 4 Discussion

The main aim of this study was to identify genomic regions under selective pressure in Angus and Hanwoo cattle utilizing imputed whole genome information. We first identified individual selection signals by four distinct methods primarily based on allele frequency and haplotype patterns. We combined individual signals to identify strong signals of selection. Finally, we identified various positional candidate genes related to beef production and quality. Overall, we observed more genomic regions and genes under selective pressure in Angus than in Hanwoo with a limited overlap of selected regions or genes between the breeds, which is consistent with large differences in breed origin, environmental habitats, divergent selection histories, breeding program objectives and ultimately, the phenotypic differences between the breeds.

Genes identified within selected genomic regions in Angus included previously known regulators of growth, body size, feed intake, reproductive performance, and immunity. For example, *PLAG1* regulates cell proliferation and its association with carcass weight and stature has been reported in several cattle breeds (Utsunomiya et al., 2013a; Takasuga, 2016; Fink et al., 2017). Similarly, *LYN*, another regulator of cell proliferation and *RPS20*, a catalyst of protein synthesis, have been associated with body weight and preweaning daily gain in Nellore (Utsunomiya et al., 2013a; Fink et al., 2017). *CHCHD7* was previously reported as significantly associated with height in Jersey and Holstein (Utsunomiya et al., 2013a; Fink et al., 2017) and with carcass weight in Wagyu cattle (Nishimura et al., 2012). Both *PLAG1* and *RPS20* have also been associated with fetal growth and calving ease (Takasuga, 2016). Several olfactory receptors were also found in significant genomic regions (e.g., *OR6C76, OR6C75, OR10A7, OR13C3, OR13C8*). The olfactory transduction pathway has been associated with feed intake as it affects the perception of odor and in turn influences food preference and consumption (Abo-Ismail et al., 2010). Olfactory receptor loci have also been identified in other selective sweep studies in cattle and there are indications of recent duplication events (Ramey et al., 2013); which suggests that olfactory receptors may be under strong selection. *TMEM68* (a cyltransferase involved in glycerolipid metabolism) and *XKR4* have been associated with growth and feed intake in Nellore (Terakado et al., 2018). *XKR4* has also been associated with subcutaneous fat in indicine and composite cattle (Porto Neto et al., 2012). TGS1 (trimethylguanosine synthase 1) has pleitropic effects in growth traits and feed efficiency (Terakado et al., 2018; Ghoreishifar et al., 2020). GDF5 (growth differentiation factor) is critical for normal skeletal development. Loss of GDF5 function results in developmental delay and a shortened appendicular skeleton (Buxton et al., 2001). PIK3CD (a component of the phosphatidylinositol-3-kinase pathway) is involved in lymphocyte signaling. Mutations in PIK3CD causes immune dysregulation and disease pathogenesis (Tangye et al., 2019). SPSB1 (splA/ryanodine receptor domain and SOCS box containing 1) is an important component of mammalian innate immune system regulation that recognizes foreign molecules derived from pathogens (Lewis et al., 2011). We also identified solute carrier genes (SLC44A1, SLC16A7, SLC25A33) which belong to a major class of transport proteins in the cell membrane and play an important role in response to metabolic states and environmental conditions (Pizzagalli et al., 2021). Various solute carrier genes were also identified in another study directly comparing zebu and taurine cattle using differential allele frequency and haplotype diversity methods (Chan et al., 2010). This strongly suggests their role in adaptation to tropical environments. ASIP (Agouti signaling protein) is a well-known gene associated with coat color pigmentation and environmental adaptation in several species (Bertolini et al., 2018).

Considering the breed’s innate characteristics and the high focus of the Hanwoo breeding program to select for increased marbling, it was reasonable to expect that some genomic regions under selection would be related to marbling score. An important region on CHR 24 was identified which contained ENSBTAG00000046153, MC2R and SETBP1 genes. The same region was also identified by composite signal in a multi breed study within a Hanwoo-specific signal (Gutiérrez-Gil et al., 2015). MC2R (adrencorticotropin receptor) and MC5R (melanocortin 5 receptor) genes belong to a family of melanocortin receptors (reviewed by Switonski et al. (2013)) that are involved in fatty acid and lipid metabolism pathways and reproduction. These genes have been previously located within a QTL region for marbling and backfat thickness and meat quality in pigs (Kováčik et al., 2012; Switonski et al., 2013). MC5R is a functional candidate for fatness in domestic animals and obesity in humans (Switonski et al., 2013) because it regulates interleukin 6 (IL6) (Jun et al., 2010) and downregulates leptin secretion (Hoggard et al., 2004) respectively resulting in increased fat deposition and increased feed intake. Based on these findings, we conclude that this selected region on CHR 24 is an important functional region for meat quality and should be further investigated in future studies in Hanwoo and/or other beef cattle.

Although it is common to focus on the genes identified in selected genomic regions, it should also be considered that much of the phenotypic diversity originates from differential regulation of gene expression by regulatory elements like promoters, enhancers, silencers, etc. (van Laere et al., 2003; Salinas et al., 2016). In this study 29.67% and 27.3% of the significant SNPs found in Angus and Hanwoo were annotated to gene coding regions, while the majority of the significant variants were located elsewhere. Similarly, Vernot et al. (2012) reported that the number of regulatory variants under selection far exceeded the number of variants in protein coding regions although their effect sizes may be small. Therefore, apart from the genes highlighted above, there may also be important regulatory elements within these significant genomic regions that play an important role in determining the phenotypic diversity of these breeds.

Detection of signatures of selection in populations can be challenging as it may be confounded with various other events in the population’s history that can lead to false positive results, e.g., population bottlenecks, migration, and genetic drift. Ascertainment bias is also a common problem in SNP data (Vitti et al., 2013). This study utilized whole genome sequence information from thousands of animals which should, to some extent, mitigate these issues. However, our study did not account for variation in the rate of recombination which may mimic the characteristics of selection signals (Haasl and Payseur, 2016). We also did not focus on other types of structural variants under selection such as copy number variants and tandem repeats which can play important biological roles. Moreover, the cutoff values used to initially filter the raw selection sweep signals across methods is largely arbitrary. For example, studies analyzing genotype data tend to adopt more liberal cutoffs of top 1% or 5% while those based on sequence data typically use a more conservative cutoff value such as the top 0.1% or 0.01%. For discovery of important QTLs or therapeutic targets, these thresholds may have downstream implications. In this study we first used 0.02% as a threshold of significance for individual selection signals just to highlight the peak genomic regions. We acknowledge this choice is subjective and these peaks were not used for any downstream analysis. Importantly, for the DCMS *p*-values we adopted a more conservative approach and used FDR cutoff of 0.05 which is widely used and acceptable in animal breeding and genetics. Candidate gene search was only performed for SNPs that passed the FDR cut-off based on the DCMS *p*-values. Theoretically, this approach should control the false positive rate in this study.

Signatures of selection can serve as a complementary method to genome-wide association studies for identification of functional variants in the genome and to provide new insights into the underlying biology of traits important for agricultural production. Since detecting selected genomic regions does not require phenotypic data, these studies can be particularly useful to identify genes for traits that are difficult or at time impossible to measure, for example, adaption to extreme environment and disease resistance. Significant genomic regions in this study may be used to select SNPs in future and test for their predictive ability. However, SNPs located in conserved genomic regions may have lower frequencies making it difficult to estimate their effects correctly and thus using them for prediction. These challenges may be overcome by overlapping results from various selection sweeps as well as GWAS particularly for traits that are known to be regulated by large effect loci. Finally, future projects comparing Hanwoo and Angus against indicine cattle breeds may also reveal candidate genes related to environmental adaptation.

## 5 Conclusion

To date, this is the largest signatures of selection study in Angus and Hanwoo beef cattle, both in terms of the density of SNPs and the number of animals per breed. We detected more selected genomic regions in Angus than in Hanwoo and the total length of genomic regions with evidence of selection was also higher in Angus. Moreover, we observed that the signatures of selection in Hanwoo and Angus are unique markedly reflecting differences in their selection history, genomic architecture and breed characteristics. More specifically, in Angus, we identified genes associated with growth, body size, feed intake, reproductive development and immunity, while in Hanwoo important genes associated with immunity, fat deposition, cholesterol metabolism, neuronal development and meat quality were identified. Future studies may help independently validate key functional genes regulating traits associated with these breeds.

## Data Availability

The data analyzed in this study is subject to the following licenses/restrictions: Parts of the data that support the findings of this study were available from the Rural Development Administration, Republic of Korea and American Angus Association. Restrictions apply to the availability of these data, which were used under license for this study. Requests to access these datasets should be directed to gondroce@msu.edu.
