# Supplementary material for: Signatures of selection in Angus and Hanwoo beef cattle using imputed whole genome sequence data
Source: Front Genet. 2024 Aug 2;15:1368710. doi: 10.3389/fgene.2024.1368710 (PMC11331311; doi:10.3389/fgene.2024.1368710)
Supplement: Supplementary file 1 [file Table1.DOCX]

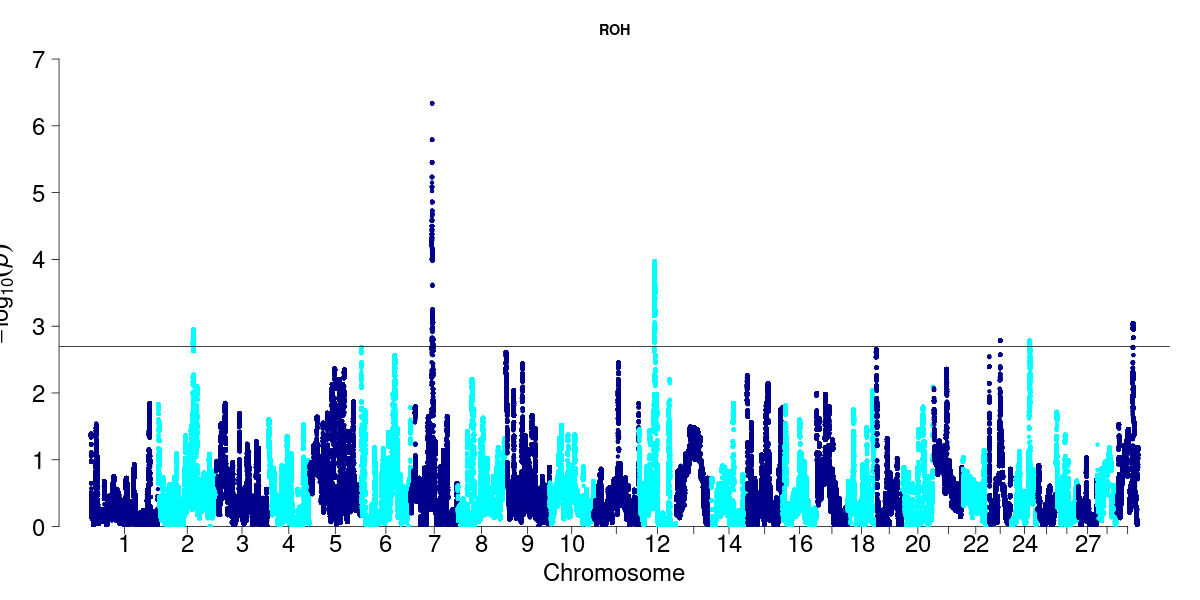


Figure S1: Plot of -log10 P values obtained from ROH analysis in Hanwoo.


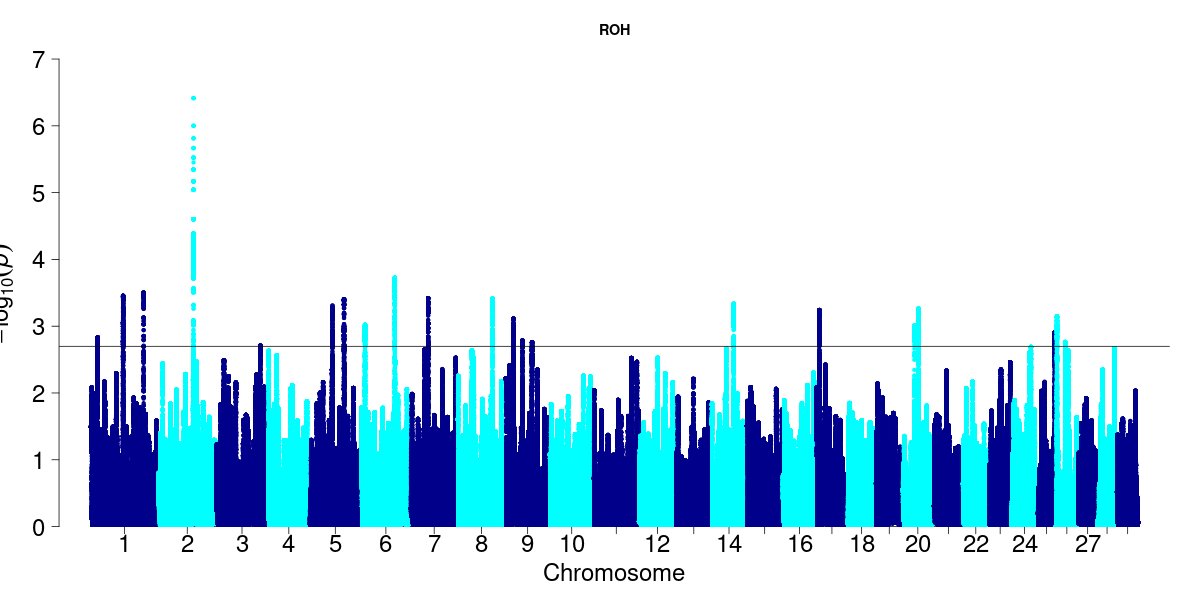


Figure S2: Plot of -log10 P values obtained from iHS analysis in Hanwoo.


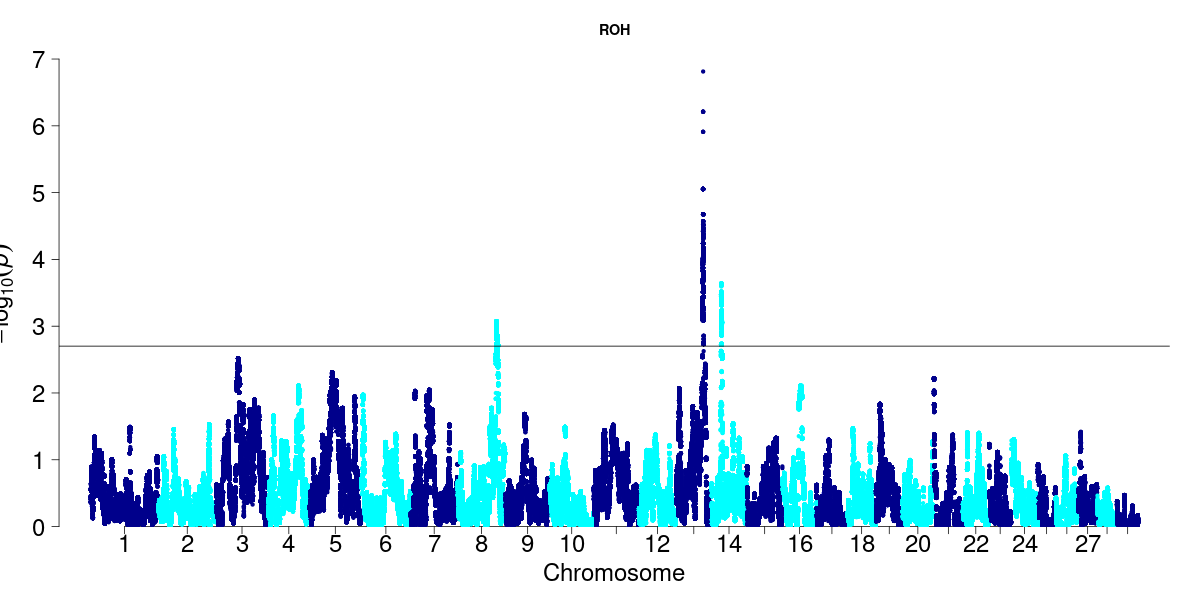


Figure S3: Plot of -log10 P values obtained from ROH analysis in Angus.


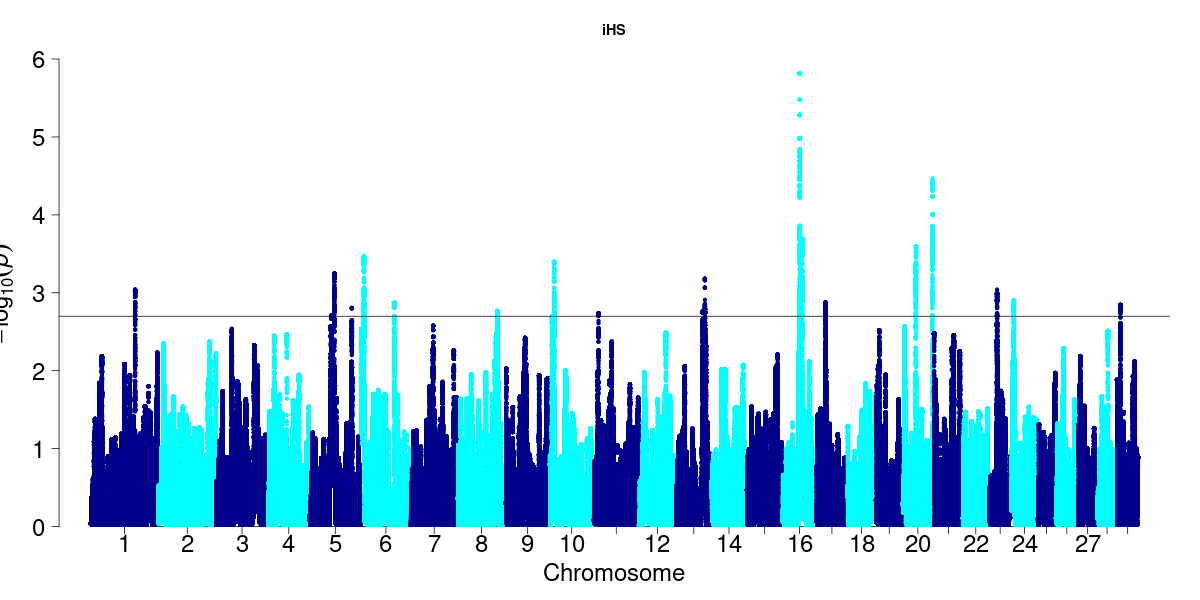


Figure S4: Plot of -log10 P values obtained from iHS analysis in Angus.


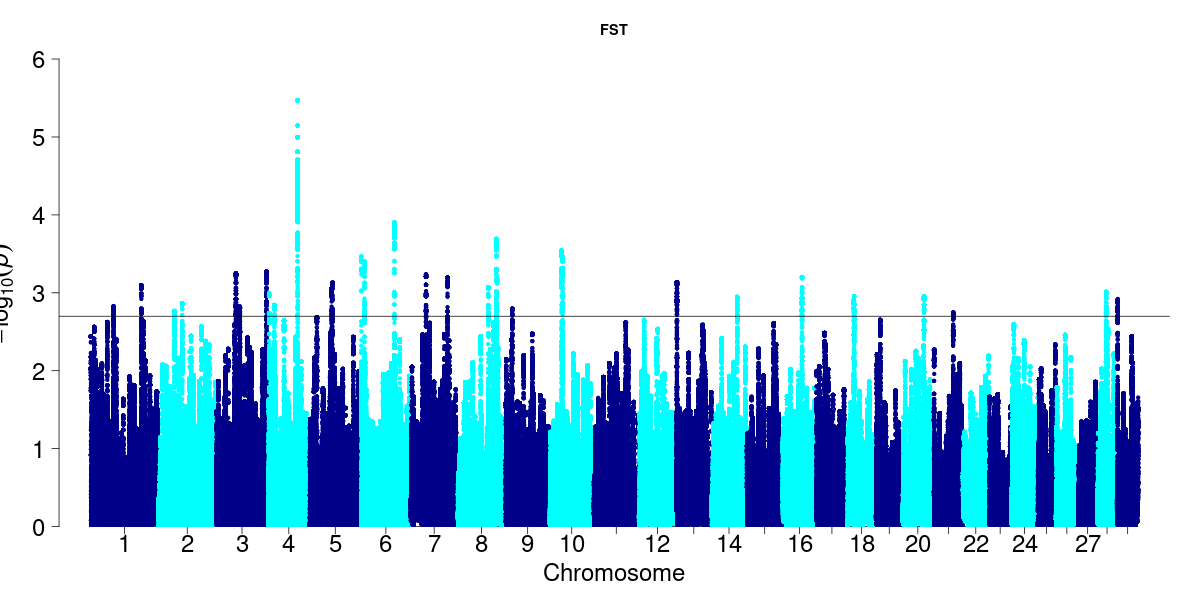


Figure S5: Plot of -log10 P values obtained from FST analysis comparing Angus and Hanwoo.


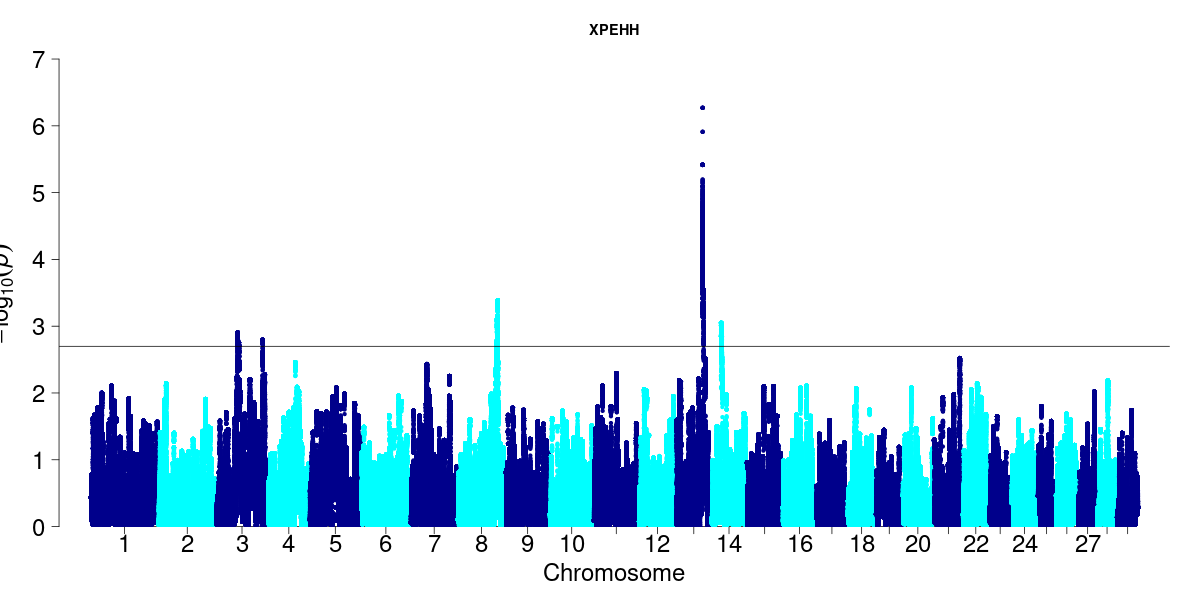


Figure S6: Plot of -log10 P values obtained from XPEHH analysis comparing Angus vs Hanwoo.
